# Standard Versus Reduced CDK4/6 Inhibitor Therapy in Elderly Patients with Metastatic Hormone Receptor-Positive, HER2-Negative Breast Cancer: An Observational Multicenter Study

**DOI:** 10.3390/jcm13237441

**Published:** 2024-12-06

**Authors:** Palma Fedele, Matteo Landriscina, Lucia Moraca, Arianna Gadaleta-Caldarola, Antonio Cusmai, Francesco Giuliani, Vincenzo Chiuri, Francesco Giotta, Antonello Pinto, Valentina Mirisola, Gennaro Gadaleta-Caldarola

**Affiliations:** 1 Oncology Unit, “Dario Camberlingo” Hospital, 72021 Francavilla Fontana, Italy; antonello.pinto@studenti.unimi.it; 2 U.O. Medical Oncology and Biomolecular Therapy, Department of Medical and Surgical Sciences, University of Foggia, 71100 Foggia, Italy; matteo.landriscina@unifg.it; 3 Oncology Unit, “Teresa Masselli Mascia” Hospital, 71100 San Severo, Italy; lucia.moraca@aslfg.it; 4 Oncology Unit, “Mons. A. R. Dimiccoli” Hospital, 70051 Barletta, Italy; arianna.gadaletac@gmail.com; 5 Oncology Unit, I.R.C.C.S. “Giovanni Paolo II”, 70124 Bari, Italy; antoniocusmai@hotmail.com (A.C.); f.giotta@oncologico.bari.it (F.G.); 6 Oncology Unit, “San Paolo” Hospital, 70123 Bari, Italy; francesco.giuliani@asl.bari.it (F.G.); gennaro.gadaleta@aslbat.it (G.G.-C.); 7 Oncology Unit, “Sacro Cuore di Gesù” Hospital, 73014 Gallipoli, Italy; vincenzo.chiuri@asl.le.it; 8 Polistudium SRL, 20121 Milan, Italy; valentinamirisola77@gmail.com

**Keywords:** CDK4/6 inhibitors, metastatic breast cancer, elderly patients, dose reduction, progression-free survival, overall survival, adverse events

## Abstract

**Background:** Cyclin-dependent kinase 4 and 6 (CDK4/6) inhibitors are the standard of care for hormone receptor (HR)+/human epidermal growth factor receptor 2 (HER2)-negative metastatic breast cancer in combination with endocrine therapy. However, the real-world efficacy and safety of standard versus reduced doses in elderly patients remain unclear. This study aims to compare the clinical outcomes of standard versus reduced doses of CDK4/6 inhibitors in elderly patients with metastatic breast cancer. **Methods:** This multicenter retrospective cohort study included 158 patients aged ≥70 years diagnosed with HR+/HER2-negative metastatic breast cancer who received either standard or reduced doses of CDK4/6 inhibitors (Ademaciclib, Ribociclib, Palbociclib) as first-line therapy. Progression-free survival (PFS), overall survival (OS), and adverse events (AEs) were evaluated. PFS and OS were estimated using the Kaplan–Meier method, and comparisons between groups were performed using a log-rank test. **Results:** Of the total population, 108 patients (68.4%) received the standard dose, and 50 patients (31.6%) received a reduced dose. The standard-dose group had significantly longer median PFS compared to the reduced-dose group (21.3 vs. 15.2 months, *p* = 0.014), while the median OS did not differ significantly (37.2 vs. 37.2 months, *p* = 0.103). Subgroup analyses revealed no significant differences in PFS or OS between standard and reduced doses for Ademaciclib and Ribociclib, while Palbociclib at standard dose showed superior PFS (21.9 vs. 12.7 months, *p* = 0.029) and OS (50.5 vs. 28.6 months, *p* = 0.026). The incidence of Grade 2–4 AEs was higher in the standard-dose group (74.2% vs. 56.8%, *p* = 0.044). **Conclusions:** Dose reduction of CDK4/6 inhibitors, particularly Ademaciclib and Ribociclib, is a viable option in elderly patients, maintaining comparable OS outcomes to standard dosing while reducing the risk of adverse events. Palbociclib at standard dose may offer superior outcomes. These findings support personalized dosing strategies to optimize efficacy and tolerability in frail or elderly patients.

## 1. Introduction

Cyclin-dependent kinase 4 and 6 (CDK4/6) inhibitors (abemaciclib, palbociclib, ribociclib) in combination with endocrine therapy (ET), such as aromatase inhibitors or fulvestrant, with or without luteinizing hormone-releasing hormone (LHRH) agonists, have become the standard treatment for hormone receptor (HR)+/human epidermal growth factor receptor 2 (HER2)-negative metastatic breast cancer, except in cases of visceral crisis [1,2,3,4]. Numerous clinical trials have demonstrated a marked improvement in progression-free survival (PFS) when CDK4/6 inhibitors are added to ET, in both premenopausal and postmenopausal women. Although PFS benefits have been consistently observed across trials, significant overall survival (OS) benefits have only been seen in certain studies [5,6,7,8,9,10,11,12].

In elderly patients, managing metastatic breast cancer is particularly complex due to the presence of comorbidities and frailty. Although current guidelines do not recommend dose reductions based on age, treatment decisions are often guided more by the patient’s chronological age than by their functional status, which may result in inappropriate treatment intensity. Frailer patients are sometimes overtreated, while those in better condition might be undertreated. Despite the fact that about one-third of breast cancer cases occur in patients aged 70 or older, this demographic is underrepresented in clinical trials. Furthermore, those elderly patients who do participate in trials tend to be fitter than the general population of older patients, creating a gap in understanding how these therapies perform in typical elderly patients [13].

A key concern regarding the use of CDK4/6 inhibitors in older patients is the risk of adverse events, which can lead to treatment delays, dose reductions, or even discontinuations. This could affect the overall effectiveness of treatment. Nevertheless, pivotal trials such as PALOMA-2, PALOMA-3, MONALEESA-3, MONALEESA-2, MONARCH-3, and MONARCH-2 have shown that CDK4/6 inhibitors provide substantial benefits in PFS and, in some cases, OS, even in older populations, though the representation of elderly patients in these studies remains limited [5,6,7,8,9,10,11,12]. More research is required to refine treatment approaches for elderly patients, ensuring a balance between efficacy and tolerability. In particular, the use of an initial reduced dose in frail patients can help reduce the risk of adverse effects hampering therapy [14].

To address these gaps in knowledge, we conducted a multicenter retrospective study to evaluate the outcomes of CDK4/6 inhibitor therapy in women aged 70 and older with HR+/HER2-metastatic breast cancer [15]. Building on the findings of the core study, this sub-analysis explores the impact of CDK4/6 inhibitor dosing—comparing standard versus reduced doses—on key clinical outcomes. The primary goal of this analysis is to assess the influence of dose on PFS, while secondary objectives include evaluating OS and treatment-related adverse events (using the National Cancer Institute Common Terminology Criteria for Adverse Events).

## 2. Methods

### 2.1. Study Design and Patients

This study is a sub-analysis of a larger retrospective cohort study involving elderly patients diagnosed with metastatic hormone receptor-positive (HR+)/HER2-negative (HER2-) breast cancer [15]. For this sub-analysis, we included patients who began first-line treatment with CDK4/6 inhibitors between 1 January 2020 and 15 May 2024, across multiple centers. Eligible patients were 70 years or older and received CDK4/6 inhibitors at either the standard dose or a reduced dose as part of their initial treatment for metastatic disease. Prior treatment for localized breast cancer was permitted.

Collected information included sociodemographic factors (age, comorbidities), clinical characteristics (performance status, Body Mass Index [BMI]), pathological data (histology, intrinsic breast cancer subtypes or molecular classification), details of metastases (location), and type of CDK4/6 inhibitor (Ademaciclib, Ribociclib, Palbociclib). Geriatric assessments were performed using the G8 score.

CDK4/6 inhibitors were administered according to the standard dosing regimens for each specific drug: Abemaciclib 150 mg bid, Ribociclib 600 mg/day, palbociclib 125 mg/day. Some patients received a reduced dose (Abemaciclib 100 mg bid, Ribociclib 400 mg/day, palbociclib 100 mg/day) according to physician discretion, taking into account factors such as age, comorbidities, and tolerance, as per physician judgment. Therapy continued until disease progression, intolerable toxicity, or death.

All data were anonymized in compliance with the European General Data Protection Regulation (GDPR) (2016/679) by assigning each patient a unique anonymous numerical code. This unique code enabled electronic linkage across different databases, ensuring patient privacy and data security. All results were presented in aggregated form, ensuring that data were not attributable to any single institution or individual. The study was reviewed and approved by the Ethics Committee of the Puglia Region.

### 2.2. Study Endpoints

The efficacy endpoints of this sub-analysis were as follows: (i) Progression-Free Survival (PFS), defined as the duration from the first administration of a CDK4/6 inhibitor to the first documented progression or death from any cause, whichever occurred first; and (ii) Overall Survival (OS), defined as the time from the first administration of a CDK4/6 inhibitor to death from any cause.

Patients without progression at the last follow-up were considered censored for PFS, and patients alive at the last follow-up were censored for both OS and PFS.

Safety was assessed by recording adverse events (AEs) related to CDK4/6 inhibitors, evaluated using the National Cancer Institute Common Terminology Criteria for Adverse Events (NCI-CTCAE) version 5.0. AEs were categorized by severity (Grades 1–4) and frequency and compared between the standard-dose and reduced-dose groups.

### 2.3. Statistical Analysis

Descriptive statistics were used to summarize the baseline characteristics of the population, including medians with interquartile ranges for continuous variables and absolute frequencies with percentages for categorical variables. The characteristics of patients receiving standard and reduced doses were compared using chi-square tests for categorical variables and *t*-tests or Mann–Whitney U tests for continuous variables.

PFS and OS were estimated using the Kaplan–Meier method and presented as median values with corresponding 95% confidence intervals (CIs). Comparisons between the two dose groups were made using the log-rank test. Statistical significance was set at a *p*-value < 0.05, and all analyses were conducted using the R statistical software platform (version 4.4.1).

## 3. Results

### 3.1. Baseline Characteristics

The study population consisted of 158 elderly patients with metastatic breast cancer undergoing treatment with CDK4/6 inhibitors. Of these, 108 patients (68.4%) received the standard dose, while 50 patients (31.6%) received a reduced dose of therapy. Table 1 depicts their baseline characteristics. A total of 48 patients were treated with Ademaciclib, 53 patients with Ribociclib, and 57 patients with Palbociclib.

Patients receiving the reduced dose were significantly older both at the time of diagnosis and at the initiation of therapy. The mean age at diagnosis for the standard-dose group was 72.7 years (±6.7), while it was 76.7 years (±5.0) for the reduced-dose group (*p* < 0.001). At the start of therapy, patients receiving the standard dose had a mean age of 75.1 years (±5.0) compared to 77.5 years (±4.6) in the reduced-dose group (*p* = 0.005). Although there was no significant difference in Body Mass Index (BMI), the mean BMI was slightly higher in the standard-dose group (27.3 kg/m^2^ vs. 25.6 kg/m^2^; *p* = 0.068).

Regarding cancer staging, patients receiving the reduced dose were significantly more likely to present with advanced-stage disease. A higher proportion of patients in the reduced-dose group were diagnosed at stage IV (82.0% vs. 62.3%; *p* = 0.029). There were no statistically significant differences in tumor subtype (luminal), presence of bone or visceral metastases, or in performance status (PS) according to the Eastern Cooperative Oncology Group (ECOG) scale between the two groups. A similar pattern was reported when analyzing the single treatment groups separately.

### 3.2. Survival in the Overall Populations

Table 2 depicts the survival data in the standard-dose group and the reduced-dose group. PFS was significantly different between the two dosing groups. Among the 158 patients, 149 (94.3%) experienced disease progression. Specifically, 100 patients (92.6%) in the standard-dose group and 49 patients (98.0%) in the reduced-dose group progressed (Chi-square *p* = 0.174). Using Kaplan–Meier survival analysis, the median PFS was significantly longer in the standard-dose group compared to the reduced-dose group: 21.3 months (95% CI 18.2–24.4) versus 15.2 months (95% CI 11.7–18.6), respectively (Log-Rank *p* = 0.014).

In terms of OS, 24 patients (15.2%) died during the study period, with 14 patients (13.0%) from the standard-dose group and 10 patients (20.0%) from the reduced-dose group (Chi-square *p* = 0.253). The median OS was not significantly different between the two groups. Both the standard dose and reduced-dose groups had a median OS of 45.2 months (Log-Rank *p* = 0.103).

### 3.3. Patients Treated with Ademaciclib

**Figure 1:**
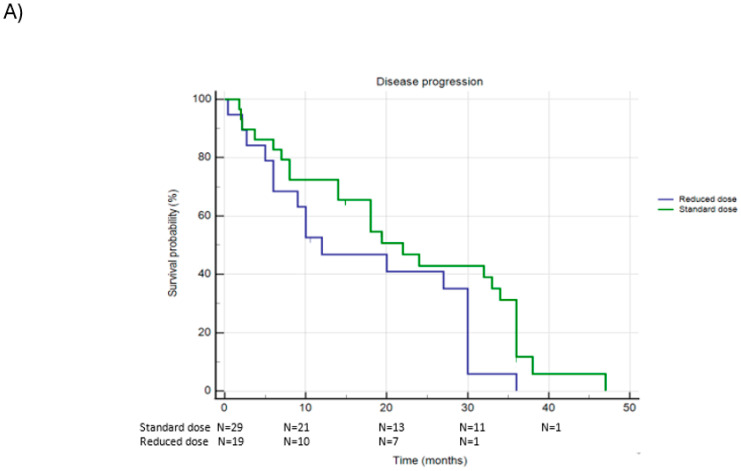

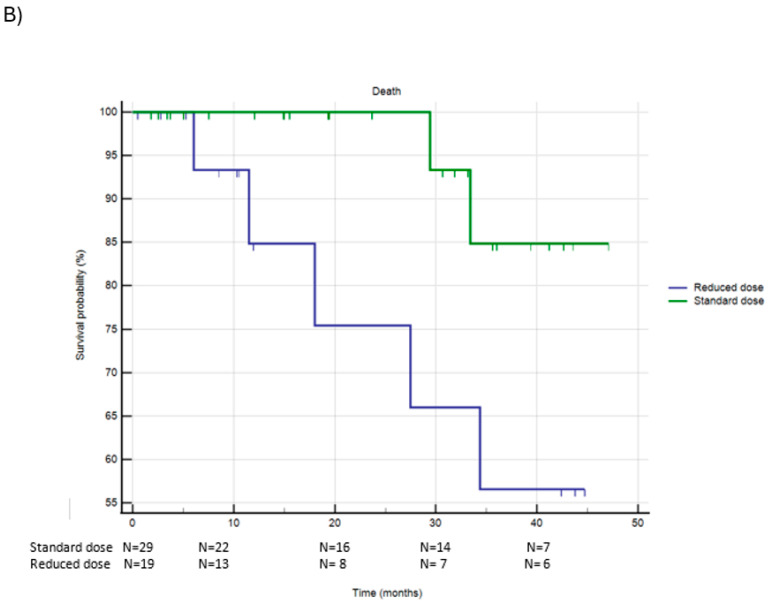
Panel (**A**): PFS in patients receiving standard dose or reduced dose of Ademaciclib; Panel (**B**): OS in patients receiving standard dose or reduced dose of Ademaciclib.

Of the 48 patients treated with Ademaciclib, 29 (60.4%) received the standard dose, while 19 patients (39.6%) were administered a reduced dose. Figure 1 and Table 3 document PFS and OS in this population.

A total of 44 patients (91.7%) experienced disease progression during treatment with Ademaciclib: 26 patients (89.7%) in the standard-dose group and 18 patients (94.7%) in the reduced-dose group progressed (Chi-square *p* = 0.538). The Kaplan–Meier analysis demonstrated that the median PFS was similar in the two groups: 22.7 months (95% CI 17.4–27.9) for the standard-dose group and 17.0 months (95% CI 11.4–22.6) for the reduced-dose group (Log-Rank *p* = 0.058).

Regarding Overall Survival (OS), 7 patients (14.6%) died in the Ademaciclib group. Of these, 2 patients (6.9%) were from the standard-dose group, and 5 patients (26.3%) were from the reduced-dose group (Chi-square *p* = 0.065). The median OS was 44.7 months (95% CI 41.7–47.7) in the standard-dose group and 34.2 months (95% CI 34.2–26.5), without any statistical difference (Log-Rank *p* = 0.050).

### 3.4. Patients Treated with Ribociclib

**Figure 2:**
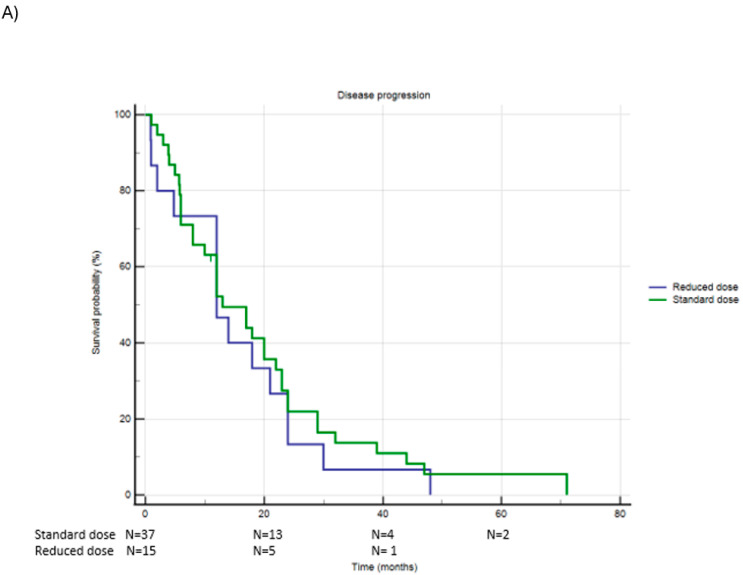

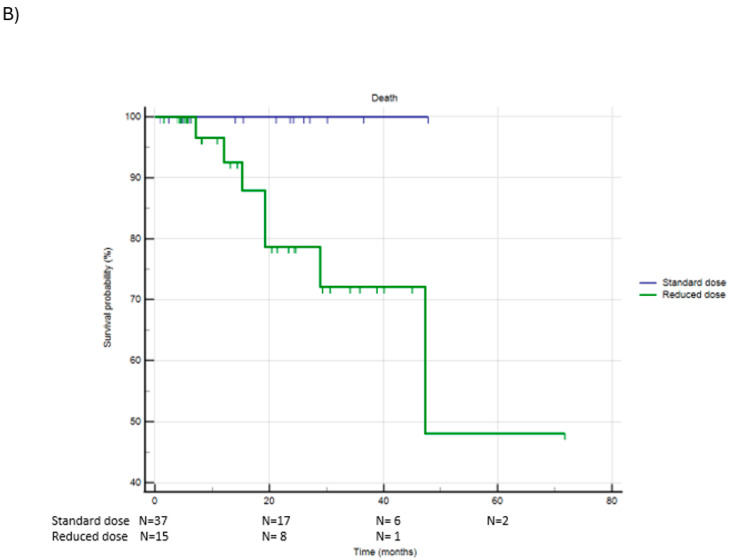
Panel (**A**): PFS in patients receiving standard dose or reduced dose of Ribociclib; Panel (**B**): OS in patients receiving standard dose or reduced dose of Ribociclib.

Of the 53 patients treated with Ribociclib, 38 (71.7%) received the standard dose, while 15 patients (28.3%) were administered a reduced dose. Figure 2 and Table 3 document PFS and OS in this population.

A total of 52 patients (98.1%) experienced disease progression during treatment with Ribociclib: 37 patients (97.4%) in the standard-dose group and all 15 patients (100%) in the reduced-dose group progressed (Chi-square *p* = 0.530). The Kaplan–Meier analysis demonstrated that the median PFS was similar between the two groups: 19.3 months (95% CI 13.7–24.8) for the standard-dose group and 15.7 months (95% CI 9.3–22.1) for the reduced-dose group (Log-Rank *p* = 0.594).

Regarding Overall Survival (OS), 7 patients (14.3%) died in the Ribociclib group, all of whom were from the standard-dose group (18.4%). No deaths occurred in the reduced-dose group (Chi-square *p* = 0.077). All deaths followed disease progression. The median OS was 51.0 months (95% CI 38.1–63.8) in the standard-dose group and 47.8 months (IC 95% 47.8–47.8) in the reduced-dose group (Log-Rank *p* = 0.103).

### 3.5. Patients Treated with Palbociclib

**Figure 3:**
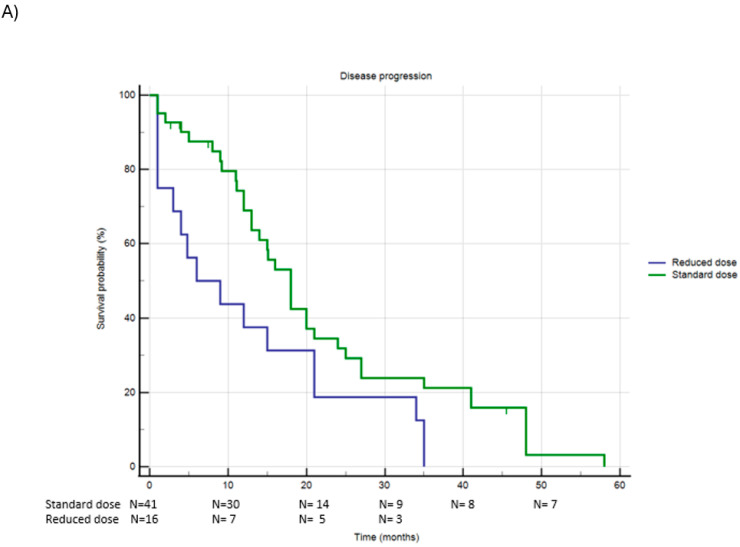

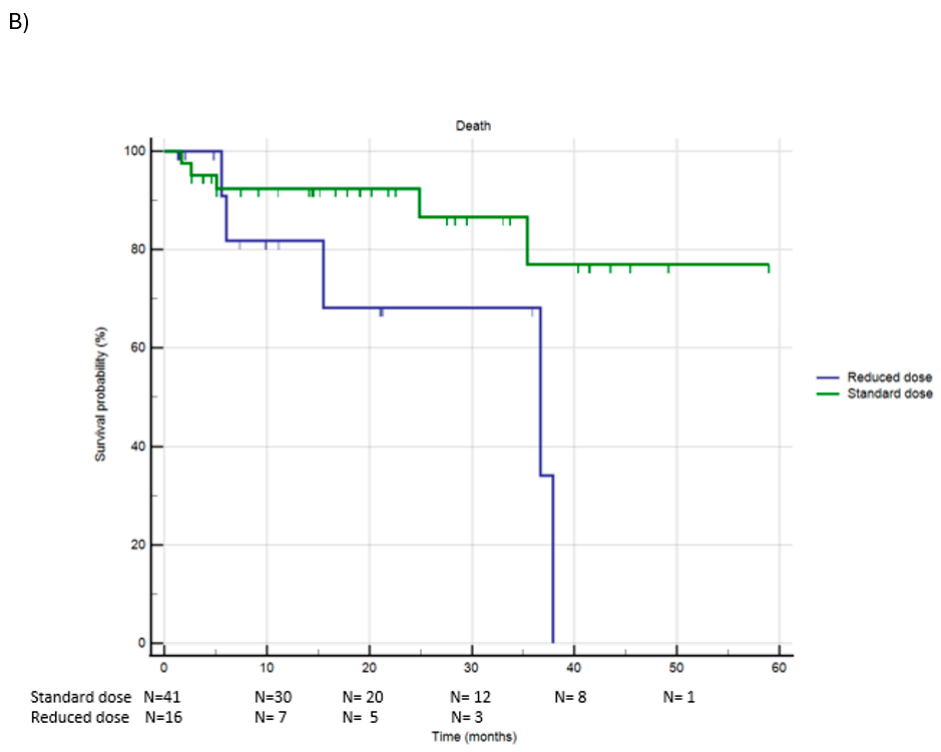
Panel (**A**): PFS in patients receiving standard dose or reduced dose of Palbociclib; Panel (**B**): OS in patients receiving standard dose or reduced dose of Palbociclib.

Of the 57 patients treated with Palbociclib, 41 (71.9%) received the standard dose, while 16 patients (28.1%) were administered a reduced dose. Figure 3 and Table 3 document PFS and OS in this population.

A total of 53 patients (93.0%) experienced disease progression during treatment with Palbociclib: 37 patients (90.2%) in the standard-dose group and all 16 patients (100%) in the reduced-dose group progressed (Chi-square *p* = 0.199). The PFS was significantly longer in the standard-dose group compared to the reduced-dose group: 21.9 months (95% CI 16.9–26.8) versus 12.7 months (95% CI 6.5–19.0), respectively (Log-Rank *p* = 0.029).

Regarding Overall Survival (OS), 10 patients (17.2%) died in the Palbociclib group. Of these, 5 patients (12.2%) were from the standard-dose group, and 5 patients (31.2%) were from the reduced-dose group (Chi-square *p* = 0.092). The median OS was significantly longer in the standard-dose group compared to the reduced-dose group: 50.5 months (95% CI 43.6–57.4) versus 28.6 months (95% CI 19.3–37.9), respectively (Log-Rank *p* = 0.026).

### 3.6. Safety and Adverse Events

Adverse events (AEs) were reported in 133 patients (84.2%) across the entire study population (Table 4 and Supplementary Material). The incidence of AEs varied between the standard-dose and reduced-dose groups, with a higher frequency of grade 1 adverse events observed in the reduced-dose group, while higher-grade events were more common in patients receiving the standard dose.

In the standard-dose group, 5 patients (4.6%) did not experience any adverse events, while 2 patients (4.0%) in the reduced-dose group reported no AEs (*p* = 0.859). Grade 1 adverse events were significantly more frequent in the reduced-dose group, occurring in 17 patients (34.0%) compared to 18 patients (16.7%) in the standard-dose group (*p* = 0.015). Conversely, when considering only grade 2, 3, and 4 adverse events, the overall incidence was significantly higher in the standard-dose group compared to the reduced-dose group (74.2% vs. 56.8%, *p* = 0.044), indicating a higher burden of moderate to severe toxicity in patients receiving the full dose of CDK4/6 inhibitors.

A total of 73 patients (46.2%) required temporary interruption of therapy due to grade 3–4 adverse events. Although more frequent in the standard-dose group, the difference in interruption rates was not statistically significant between the groups (50.9% in the standard-dose group vs. 36.0% in the reduced-dose group, *p* = 0.081). Permanent discontinuation of therapy occurred in 46 patients (29.1%) due to worsening of the performance status or patient’s refusal to continue the therapy in the absence of progression; there was no significant difference in frequency between the standard-dose group (28.7%) and the reduced-dose group (30.0%) (*p* = 0.868).

## 4. Discussion

CDK4/6 inhibitors are now firmly established as a key treatment option for HR+/HER2-negative metastatic breast cancer, with significant benefits demonstrated in clinical trials. However, the real-world application of these therapies, particularly in elderly patients, is less well understood. In this sub-analysis of a previous study [15], we investigated the outcomes of elderly patients treated with either standard or reduced doses of CDK4/6 inhibitors, with a specific focus on PFS, OS, and adverse events.

Optimizing the dosage of oncological therapies in subgroups of patients may prevent toxicity, premature discontinuation, and impaired quality of life without reducing efficacy. This may be especially important for subgroups of patients, and research is ongoing to detect relevant criteria and educate academics, professionals, regulatory authorities, and patients, to the importance of correct dosing, as promoted by the FDA Optimus project (https://www.fda.gov/about-fda/oncology-center-excellence/project-optimus (accessed on 7 October 2024)) [16].

Our findings highlight significant differences in PFS between the two dosing groups across the total population, with patients receiving the standard dose experiencing longer PFS than those on the reduced dose (21.3 months vs. 15.2 months, *p* = 0.014). However, when examining individual CDK4/6 inhibitors, notably Ademaciclib and Ribociclib, the differences between standard and reduced dosing were not significant. For Ademaciclib, the median PFS was similar between the two dose groups and OS was likewise comparable. A similar pattern was observed for Ribociclib, where PFS and OS were also similar between the standard- and reduced-dose groups. These results suggest that for some CDK4/6 inhibitors, dose reduction may not compromise efficacy in terms of PFS or OS, particularly for older patients who may be more vulnerable to adverse events.

This finding is particularly relevant given the concerns surrounding the tolerability of CDK4/6 inhibitors in elderly populations. In clinical practice, physicians often reduce the dose of these agents based on patient age, performance status, and comorbidities to minimize toxicity. In our study, patients in the reduced-dose group were older and had more advanced disease at diagnosis, which likely reflects these real-world clinical decisions [14]. Despite these adjustments, the similar OS between the two dosing groups for Ademaciclib and Ribociclib indicates that dose reductions can be made without significantly affecting survival outcomes.

On the other hand, patients treated with Palbociclib showed a more distinct benefit from the standard dose, with significantly longer PFS (21.9 months vs. 12.7 months) and OS (50.5 months vs. 28.6 months) compared to the reduced-dose group. This suggests that for some CDK4/6 inhibitors, such as Palbociclib, maintaining the standard dose may be more important for optimizing patient outcomes, particularly in terms of survival [13].

In terms of safety, the incidence of adverse events (AEs) was significantly higher in the standard-dose group, with moderate to severe (Grade 2–4) AEs occurring more frequently (74.2% vs. 56.8%). This aligns with previous studies suggesting that dose reduction can help mitigate toxicity in elderly or frail patients. However, it is reassuring that the reduction in AEs did not lead to a significant compromise in OS for patients treated with reduced doses of Ademaciclib and Ribociclib. These results suggest that individualized dosing strategies can be effective in maintaining a balance between treatment efficacy and safety, particularly in frail or elderly patients who may not tolerate full-dose therapy.

This study has several limitations. As a retrospective analysis, the potential for confounding factors and selection biases cannot be entirely excluded. Additionally, this study was conducted at a limited number of centers, which may limit the generalizability of the findings to broader populations. Nonetheless, the study provides valuable insights into the real-world use of CDK4/6 inhibitors in elderly patients, particularly regarding the safety and efficacy of dose reductions. Further studies, with longer-term observation and evaluation of quality of life, could better define the suitability for dose reduction and criteria for patient selection.

## 5. Conclusions

In conclusion, our sub-analysis suggests that dose reduction of CDK4/6 inhibitors, particularly Ademaciclib and Ribociclib, can be a viable strategy for elderly patients with HR+/HER2-negative metastatic breast cancer without compromising overall survival. While dose reduction may lead to shorter PFS in some cases, the comparable OS outcomes suggest that reduced doses can still be effective in maintaining long-term disease control, especially in frail patients or those with comorbidities. These findings support the need for personalized dosing strategies in clinical practice, optimizing treatment tolerability while preserving therapeutic efficacy.

## Figures and Tables

**Table 1:** Baseline characteristics of the study population.

Characteristic	Standard Dose (*N* = 108)	Reduced Dose (*N* = 50)	*p*-Value
Age at diagnosis (years)	72.7 (±6.7)	76.7 (±5.0)	<0.001
Age at therapy initiation (years)	75.1 (±5.0)	77.5 (±4.6)	0.005
BMI (kg/m^2^)	27.3 (±5.4)	25.6 (±5.0)	0.068
Stage at diagnosis			0.029
*-Stage I*	4 (3.8%)	1 (2.0%)	
*-Stage II*	9 (8.5%)	5 (10.0%)	
*-Stage III*	27 (25.5%)	3 (6.0%)	
*-Stage IV*	66 (62.3%)	41 (82.0%)	
Luminal subtype	43 (40.2%)	25 (50.0%)	0.249
Metastases			0.295
*-Bone*	34 (31.5%)	20 (40.0%)	
*-Visceral*	74 (68.5%)	30 (60.0%)	
G8 scale	14.1 (±1.6)	13.6 (±1.9)	0.066
PS ECOG			0.077
*-0*	30 (27.8%)	10 (20.0%)	
*-1*	57 (52.8%)	22 (44.0%)	
*-2*	21 (19.4%)	18 (36.0%)	

**Table 2:** PFS and OS in the overall population.

Survival Parameter	Standard Dose (*N* = 108)	Reduced Dose (*N* = 50)	*p*-Value
PFS (months)	21.3 (95% CI 18.2–24.4)	15.2 (95% CI 11.7–18.6)	0.014
Number of events (progression)	100 (92.6%)	49 (98.0%)	0.174
OS (months)	45.2 (95% CI 40.2–49.2)	45.2 (95% CI 39.0–51.4)	0.103
Number of deaths	14 (13.0%)	10 (20.0%)	0.253

**Table 3:** Survival data for patients treated with Ademaciclib, Ribociclib, and Palbociclib at standard or reduced dose.

Drug	Dose	Patients (*N*)	PFS (Months)	PFS Events (%)	OS (Months)	OS Events (%)	PFS *p*-Value	OS *p*-Value
Ademaciclib	*Standard Dose*	29	22.7 (95% CI 17.4–27.9)	26 (89.7%)	44.7 (95% CI 41.7–47.7)	2 (6.9%)	0.058	0.050
	*Reduced Dose*	19	17.0 (95% CI 11.4–22.6)	18 (94.7%)	34.2 (95% CI 34.2–26.5)	5 (26.3%)		
Ribociclib	*Standard Dose*	38	19.3 (95% CI 13.7–24.8)	37 (97.4%)	51.0 (95% CI 38.1–63.8)	7 (18.4%)	0.594	0.103
	*Reduced Dose*	15	15.7 (95% CI 9.3–22.1)	15 (100%)	Not reached	0 (0%)		
Palbociclib	*Standard Dose*	41	21.9 (95% CI 16.9–26.8)	37 (90.2%)	50.5 (95% CI 43.6–57.4)	5 (12.2%)	0.029	0.026
	*Reduced Dose*	16	12.7 (95% CI 6.5–19.0)	16 (100%)	28.6 (95% CI 19.3–37.9)	5 (31.2%)		

**Table 4:** Summary of adverse events in the standard-dose and reduced-dose groups.

Grade of Adverse Events	Standard Dose (*N* = 108)	Reduced Dose (*N* = 50)	*p*-Value
No adverse events	5 (4.6%)	2 (4.0%)	0.859
Grade 1	18 (16.7%)	17 (34.0%)	0.015
Grade 2	30 (27.8%)	14 (28.0%)	0.977
Grade 3	35 (32.4%)	11 (22.0%)	0.182
Grade 4	1 (0.9%)	0 (0%)	0.496
Grade 2, 3, or 4	66 (74.2%)	25 (56.8%)	0.044
Temporary interruption	55 (50.9%)	18 (36.0%)	0.081
Permanent discontinuation	31 (28.7%)	15 (30.0%)	0.868

## Data Availability

All data are available from the Corresponding Author upon reasonable request.

## Supplementary Materials

The following supporting information can be downloaded at: https://www.mdpi.com/article/10.3390/jcm13237441/s1.
