# Supplementary material for: Standard Versus Reduced CDK4/6 Inhibitor Therapy in Elderly Patients with Metastatic Hormone Receptor-Positive, HER2-Negative Breast Cancer: An Observational Multicenter Study
Source: J Clin Med. 2024 Dec 6;13(23):7441. doi: 10.3390/jcm13237441 (PMC11641986; doi:10.3390/jcm13237441)
Supplement: Supplementary file 1 [file jcm-13-07441-s001.zip › jcm-3274015-supplementary.pdf]

Supplementary material

Adverse events in the overall population and in different treatment groups.

**Adverse Events**	**Standard Dose (N = 108)**	**Reduced Dose (N = 50)**	**p-value**
Neutropenia	21 (19.4%)	11 (22.0%)	0.711
Asthenia	6 (5.6%)	7 (14.0%)	0.073
Diarrhea	7 (6.5%)	0	0.098
Cardiac disorders	2 (1.9%)	0	1.000
Anemia	6 (5.6%)	1 (2.0%)	0.433
Thrombocitopenia	2 (1.9%)	0	1.000
Alopecia	0	1 (2.0%)	0.318

Grade of Adverse Events	Ademaciclib Standard Dose (N = 29)	Ademaciclib Reduced Dose (N = 19)	p-value
No adverse events	1 (3.4%)	1 (5.3%)	1.000
Grade 1	5 (17.2%)	7 (36.8%)	0.129
Grade 2	12 (41.4%)	4 (21.1%)	0.148
Grade 3	11 (37.9%)	6 (31.6%)	0.656
Grade 2, 3, or 4	23 (82.1%)	10 (55.6%)	0.053
Temporary interruption	20 (69.0%)	8 (42.1%)	0.068
Permanent discontinuation	10 (34.5%)	7 (36.8%)	0.869

**Grade of Adverse Events**	**Ribociclib Standard Dose (N = 38)**	**Ribociclib Reduced Dose (N = 15)**	**p-value**
No adverse events	12 (31.6%)	3 (20.0%)	0.404
Grade 1	6 (15.8%)	4 (26.7%)	0.367
Grade 2	7 (18.4%)	6 (40.0%)	0.103
Grade 3	10 (26.3%)	2 (20.0%)	0.314
Grade 4	1 (2.6%)	0	1.000
Grade 2, 3, or 4	18 (69.2%)	8 (66.7%)	0.876
Temporary interruption	17 (44.7%)	4 (26.7%)	0.230
Permanent discontinuation	10 (26.3%)	1 (6.7%)	0.116

Grade of Adverse Events	Palbociclib Standard Dose (N = 41)	Palbociclib Reduced Dose (N = 16)	p-value
No adverse events	6 (14.6%)	2 (12.5%)	0.836
Grade 1	7 (17.1%)	6 (37.5%)	0.102
Grade 2	11 (26.8%)	4 (25.0%)	0.889
Grade 3	14 (34.1%)	3 (18.8%)	0.258
Grade 2, 3, or 4	25 (71.4%)	7 (50.0%)	0.159
Temporary interruption	18 (43.9%)	6 (37.5%)	0.663
Permanent discontinuation	11 (26.8%)	7 (43.7%)	0.221
